# Movement disorders in patients with Rett syndrome: A systematic review of evidence and associated clinical considerations

**DOI:** 10.1111/pcn.13299

**Published:** 2021-10-21

**Authors:** Jatinder Singh, Evamaria Lanzarini, Nardo Nardocci, Paramala Santosh

**Affiliations:** ^1^ Department of Child and Adolescent Psychiatry Institute of Psychiatry, Psychology and Neuroscience, King's College London London UK; ^2^ Centre for Interventional Paediatric Psychopharmacology and Rare Diseases South London and Maudsley NHS Foundation Trust London UK; ^3^ Centre for Personalised Medicine in Rett Syndrome, Institute of Psychiatry, Psychology and Neuroscience King's College London London UK; ^4^ Child and Adolescent Neuropsychiatry Unit Infermi Hospital Rimini Italy; ^5^ Department of Paediatric Neurology Fondazione IRCCS Istituto Neurologico “Carlo Besta” Milan Italy

**Keywords:** dystonia, movement disorders, mutations, Rett syndrome, stereotypies

## Abstract

**Aim:**

This systematic review identified and thematically appraised clinical evidence of movement disorders in patients with Rett syndrome (RTT).

**Method:**

Using PRISMA criteria, six electronic databases were searched from inception to April 2021. A thematic analysis was then undertaken on the extracted data to identify potential themes.

**Results:**

Following the thematic analysis, six themes emerged: (i) clinical features of abnormal movement behaviors; (ii) mutational profile and its impact on movement disorders; (iii) symptoms and stressors that impact on movement disorders; (iv) possible underlying neurobiological mechanisms; (v) quality of life and movement disorders; and (vi) treatment of movement disorders. Current guidelines for managing movement disorders in general were then reviewed to provide possible treatment recommendations for RTT.

**Conclusion:**

Our study offers an enriched data set for clinical investigations and treatment of fine and gross motor issues in RTT. A detailed understanding of genotype–phenotype relationships of movement disorders allows for more robust genetic counseling for families but can also assist healthcare professionals in terms of monitoring disease progression in RTT. The synthesis also showed that environmental enrichment would be beneficial for improving some aspects of movement disorders. The cerebellum, basal ganglia, alongside dysregulation of the cortico‐basal ganglia‐thalamo‐cortical loop, are likely anatomical targets. A review of treatments for movement disorders also helped to provide recommendations for treating and managing movement disorders in patients with RTT.

As a complex neurodevelopmental disorder that begins in early childhood, Rett syndrome (RTT) presents with a range of symptoms, including autonomic, gastrointestinal, and neuropsychiatric disturbances. Movement disorders are a significant clinical concern, and their broad spectrum makes treatment and management in RTT challenging. Some of the abnormal movement disorders in RTT, such as hand stereotypies and gait disorders, form part of the essential diagnostic criteria for classical RTT alongside other supportive criteria such as bruxism and abnormal muscle tone.^1^

Emotional, behavioral, and autonomic dysregulation (EBAD) can emerge with a wide range of symptoms and makes the treatment of RTT challenging. We have previously suggested that the behavioral component of EBAD can be exacerbated by abnormal movement behaviors.^2^ Behavioral difficulties also seem to be associated with worse outcomes in patients with RTT.^3^ Managing the behavioral and emotional components of EBAD by targeting specific movement behaviors in patients with RTT could potentially improve the quality of life (QoL) in this patient group by providing potentially important clinical information to understand the patients’ needs better. Therefore, it is essential to systematically review movement disorders in patients with RTT because it would allow further exploration of the broad nature of movement disorders in patients with RTT.

Recent studies have explored instruments to evaluate gross motor and musculoskeletal deficits in patients with RTT^4^ and provided perspectives of hand functioning in females with RTT.^5^ However, while movement and motor disorders have been described in syndromic autism using systematic methods^6^ and in RTT via a narrative review,^7^ as far as we are aware, no study has undertaken a systematic review of movement disorders in patients with RTT with the intention of identifying themes to further understand the clinical impact of this movement impairment and whether the emerging information would be clinically relevant and useful when assisting in the management of patients. The purpose of this review was, therefore to: (i) undertake a robust systematic review of studies examining movement disorders in patients with RTT; (ii) use a thematic analysis approach to identify potential themes; and (iii) determine whether the information from these themes could be adopted by clinicians, therapists, and other healthcare professionals to improve the QoL of this patient group and disseminate these findings to the broader RTT community.

## Methods

### Search strategy

#### Primary search strategy

The methodology for the systematic review followed the PRISMA criteria.^8^, ^9^ Two authors (J.S. and E.L.) independently and blindly searched the following databases: PubMed, Scopus, Cochrane, PsycINFO, Embase, and Web of Science in February 2021. As described, a truncation symbol (*) was used to capture as much of the literature as possible. To ensure that the primary searching captured as much literature as possible relevant to movement disorders in patients with RTT, the search strategy was focused on stereotypies. We reasoned that because nearly all patients with RTT have stereotypies^10^, ^11^, ^12^ that co‐occur alongside other abnormal movement disorders, the search strategy would be expected to detect most of the studies of abnormal movements in patients with RTT. To extend this primary search strategy, the first author (J.S.) searched the references from the reference list (snowballing) of studies to see whether any more eligible studies regarding movement disorders in RTT could be traced. Snowballing is a useful strategy for extending systematic reviews ensuring the best possible coverage of the literature.^13^

To reduce search strategy bias, we used the principles adopted for our previous evidence synthesis.^14^ First, both the first (J.S.) and second (E.L.) author independently undertook the PRISMA systematic review in a blinded manner. Second, the consensus agreement of eligible articles was based on an agreement between J.S. and E.L., and if a consensus could not be reached, the senior author (P.S.) was consulted.

#### Secondary searches

To supplement the primary search strategy, the first author (J.S.) also conducted an additional search of the PubMed, Scopus, Cochrane, PsycINFO, Embase, and Web of Science databases in April 2021 with the specific search terms of dystonia, Parkinsonism, bruxism, spasticity, tremor, and ataxia in RTT. This secondary search was also reviewed by the second author (E.L.) and a consensus agreement was reached on the additional articles that were included. A scoping review on the treatment of movement disorders was performed by the first author (J.S.) and reviewed by the other authors.

### Search terms

#### Primary search terms

The search of the databases used the following keywords:

(Rett syndrome OR MECP2) AND (stereotypies*)

#### Secondary search terms

(Rett syndrome OR MECP2) AND (dystonia)

(Rett syndrome OR MECP2) AND (Parkinson*)

(Rett syndrome OR MECP2) AND (bruxism)

(Rett syndrome OR MECP2) AND (spasticity)

(Rett syndrome OR MECP2) AND (tremor*)

(Rett syndrome OR MECP2) AND (ataxia*)

### Population characteristics

All records within the databases that reported studies in RTT were searched.

### Intervention

These included all records that mentioned or reported on movement disorders/impairments.

### Eligibility criteria

The following eligibility criteria were used.

Inclusion criteria:

- Complete records/articles in peer‐reviewed academic/scientific journals and electronically available.
- All investigations/reports performed in humans.

Exclusion criteria:

- Records/articles not in English language and not available using electronic sources.
- Review articles, book chapters, single case reports/studies, commentaries, conference abstracts, dissertations, letters to the editor, clinical trial protocols, and preprints.

### Extraction of data and thematic analysis

Data extraction and thematic analysis were performed as previously described.^14^ The first author (J.S.) did the manual coding for the thematic analysis, which was independently reviewed by the second author (E.L.). A consensus on the themes was then reached, and the final themes that emerged were based on group consensus between all of the authors of the study. Microsoft Excel software 2016 was used to show the frequencies of the themes that arose.

## Results

The PRISMA (Supplementary Information S1) identified 690 records, and, after duplicates were removed, 230 records were screened. Following the screening process that excluded 34 records, 196 records were assessed against the eligibility criteria. This procedure eliminated 171 records, and 25 articles were eligible. After consensus agreement between the first (J.S.) and second (E.L.) authors, a further seven articles were deemed eligible, and one study^15^ was also included after snowball searching. The secondary screening of records within the databases using specific search terms identified 10 additional articles (Supplementary Information S2). In total, 43 full‐text articles were included in the analysis. The data extraction from these 43 articles is shown in Table 1.

**Table 1:** Summarized information from the eligible studies

Source	Demographics	Clinical characteristics	Assessment methods	Relevant movement impairment evidence
^10^Stallworth *et al*. (2019)	Females with RTT (1074) from the RTT Natural History Study Patients with classical RTT comprised the largest group (*n* = 922)	All study participants had a confirmed clinical diagnosis and/or mutation in *MECP2* Of the 1074 patients with RTT, 922 had classical RTT, 75 had atypical severe RTT, and 77 had atypical mild RTT Patients with classical RTT were followed for an average of 4.22 years	Standardized assessments (CSS and MBA) at baseline and analyses of longitudinal data	Hand stereotypies were reported in all (100%) patients with classical RTT, in 97.3% with atypical severe RTT, and in 96.1% with atypical mild RTT Hand mouthing and clapping/tapping was more commonly reported than hand wringing/washing Clinical severity was found to be worse with decreased hand function, and while hand use lowered over time, the frequency of hand stereotypies was noted to remain unchanged, i.e. remained unchanged and was elevated In the majority of patients, regression appeared before hand stereotypies but one‐third appeared with early onset Increases in bradykinesia and hypertonia were suggested to play a role in hand functioning
^11^Vignoli *et al*. (2012)	Females (≥14 years) were split into three age groups (14–20 years, 21–29 years, and >29 years)	Questionnaire sent to members of the Italian Association for RTT The Kerr score was used to evaluate disease severity	Questionnaire study	Stereotypies (hand) were present in nearly all patients (98%), while only 20% could independently walk Nearly all (96%) patients had musculoskeletal problems, with scoliosis (83%) being the most frequent followed by spasticity (51%) and joint deformities (36%). Joint deformities also appeared to become worse over time In terms of movement problems, the study findings indicated that stereotypies and hand functioning remained stable over time. Musculoskeletal problems worsened and continued into adulthood In the sample, older patients with some specific mutations (R294X and R133C) and C‐terminal deletions were clinically less severe
^12^Carter *et al*. (2010)	Cohort (*n* = 144) was from the ARSD	Patients were categorized according to genotype	Video data	The findings showed that nearly all patients had hand stereotypies (94.4%) Among the 15 categories evaluated, midline wringing was noted in 60% of cases In patients younger than 8 years, clapping and mouthing was more frequent, while in patients older than 19 years, wringing was more common In the 15 categories explored, the study indicated that there was no obvious relationship between hand stereotypies and genotype
^15^Humphreys and Barrowman (2016)	Fifty‐one patients ranging from 2 years 5 months to 54 years of age	Patients who were included were *MECP2* mutation positive Classification was also based on mutation type (truncating [*n* = 25] or missense [*n* = 22]), whether patients were ambulatory or were able to speak	Rigidity was assessed using the RTT rigidity distribution score	The study showed that rigidity was observed in 84.3% of patients (43 of 51). The onset was rapid, appearing at 3 years and tended to increase with age The topography began with ankle, legs, arms neck, and face No statistically significant difference in rigidity score was seen between those with truncating and missense mutations Patients who could walk had lower rigidity scores when compared with those who were less able to walk The authors suggested that Parkinsonian‐like rigidity is common in RTT
^16^Young *et al*. (2020)	Fourteen females with RTT aged (±SD) 9.2 ± 5.4 years	Each participant had a confirmed diagnosis according to Neul *et al*. (2010)^†^and a mutation in *MECP2* All patients were ambulatory	Videotape observations during overground and treadmill walking	Freezing of gait appeared to be the most frequently occurring behavior and was deemed to be an important characteristic of walking in patients with RTT Video together with overground and treadmill observation can be useful to assess ataxic gait in patients with RTT who are ambulatory Stereotypic behaviors were not observed in patients during freezing of gait and could suggest independent neural mechanisms This information can be useful for clinicians to assess the individual's gait regression over time and can provide further understanding of gross motor dysfunction in these patients
^17^Dy *et al*. (2017)	Study participants (*n* = 27) were part of the phase II RCT with mecasermin Average age was 6 years 4 months	All study participants met the diagnostic criteria for RTT Assessments were done before administration of active agent or placebo	Clinical assessment and video recording	The study indicated that hand stereotypies in patients with RTT are heterogenous and more robust objective measures are needed such as actigraphy It was also suggested that machine learning could also be able to classify movement patterns regarding abnormal movements in RTT
^18^Cianfaglione *et al*. (2016)	Ten individuals aged between 5 and 32 years (median: 12.5 years)	Of the 10 patients, nine had classical RTT and one had atypical RTT	Questionnaire assessment and video observational data from parents, teachers, and carers	Self‐injury occurred in six of the patients (five with classical and one with atypical) Environment was considered to be important in modifying the behavior of patients with RTT, especially for self‐injury but not for hand stereotypies
^19^Quest *et al*. (2014)	Study of five female patients with RTT (mean age: 17.8 years [range: 4 to 47 years])	All patients had a clinical diagnosis of classical RTT according to Neul *et al*. (2010)^†^criteria	Behavioral observations using video	The study indicated that there were no observed differences in stereotyped hand behaviors during the high and low stress conditions for any of the patients More negative signs were seen during high stress conditions as indicated by differences in the domains of face, vocalize, and tremble
^20^Nissenkorn and Ben‐Zeev (2013)	Case study of five patients with RTT ranging from 3 to 9 years of age	Five females with RTT and unilateral repetitive hand movements were noted from a sample of 64 patients with 24‐h video‐EEG recordings	Video‐EEG recording	This study identified a unique set of unilateral hand movements that was associated with centrotemporal spikes on EEG This behavior was not thought to be epileptic but rather the EEG spikes were caused by either somatosensory or motor potentials most likely originating from the somatosensory, motor cortex, or subcortical areas of the brain in patients with RTT
^21^Goldman and Temudo (2012)	The study included 20 patients with RTT (mean age: 60 months [range: 36 to 96 months]) and 20 individuals with ASD (mean age: 68 months [range: 33 to 98 months])	Patients met the RTT diagnostic criteria Patients with ASD had a preschool diagnosis based on *DSM‐III‐R* criteria	Video observations	Behavioral hand stereotypies could be distinguished between children with RTT when compared with those with ASD In RTT, the hand stereotypies were said to be complex and localized to the body midline and involved mouthing. In comparison, in children with ASD, the hand stereotypies were said to be simple and intermittent and usually included objects It was suggested that there could be abnormal basal ganglia circuitry involvement together with impaired thalamic motor input
^22^Temudo *et al*. (2011)	Patients with RTT (*n* = 87) with (group I) and without (group II) a molecular diagnosis Median age of group I (*n* = 59) was 7.6 years (range: 4.1–14.3 years) and for group II (*n* = 28) was 7.8 years (range: 5.1–12.7 years)	Patients were diagnosed according to clinical criteria (Hagberg *et al*., 2002)^‡^ Patients had either the classical (58.6%) or variant form (41.4%) Of the 59 patients with a molecular diagnosis (group I), 26 had missense mutations and 33 had truncating mutations	Video analysis and observation by a pediatric neurologist Blood samples for genomic DNA extraction X‐chromosome inactivation assays	Ataxia, number of stereotypies per patient, rigidity, and ataxic gait were statistically significant in group I when compared with group II. Dystonia was more frequent for patients in group II but was not significant (*P* = 0.458). In patients with a confirmed molecular diagnosis (group I), rigidity and dystonia were more common in those with truncating mutations. There was, however, no obvious difference in the number of stereotypies in patients with either missense or truncating mutations Perinatal data showed that patients in group I presented with a higher frequency of abnormal delivery (28.8%) when compared with group 2 (21.4%). Moreover, there was a higher frequency of abnormal delivery in patients with truncating (33.3%) compared with those with missense (23.1%) mutations. Neither of these findings reached statistical significance The authors suggest that organic features of impaired movement disorders are driven by changes in brainstem and cerebellar structures that could explain the differences in the phenotypic picture
^23^Downs *et al*. (2010)	Cross‐sectional study comprising 144 females with RTT from the ARSD Mean age: 14 years 10 months (range: 2–31 years 10 months)	All patients had a clinical diagnosis of RTT Of the 144 females, 110 were found to have an *MECP2* mutation	Video observations and parent‐reported data together with modified Kerr scores, WeeFIM, ambulation status, and number of hand stereotypies	It was indicated that patients with the p.R168X mutation in comparison to those with p.R133C or p.R294X mutations had the worst hand function Patients older than 19 years had worse hand function than those younger than 8 years When controlling for age and mutation, there was a significant association between mobility and hand function Positive environmental reinforcement could play a role in managing hand stereotypies
^24^Fabio *et al*. (2009)	Ten females with RTT aged between 5 and 26 years	All patients had a diagnosis of RTT and analyses of their *MECP2* mutation	Assessment scales and video recording of the overselectivity test	Females tended to learn more quickly when their stereotypies were contained compared with those whose stereotypies were not contained Stereotypies can be reduced when sensory stimulation is present
^25^Vignoli *et al*. (2009)	Study cohort consisted of 12 patients with RTT with a mean age of 18.6 years (range: 14–31 years)	The cohort was selected from 30 patients 14 years and older and *MECP2* mutation positive	Parental interviews, video observations, and review of clinical information	In the 12 patients, the mean onset age of stereotypies was 19.4 months and were maintained during disease progression across the lifespan In nine of 12 patients, hand functioning was lost a few months after onset of stereotypies Hand stereotypies did not occur during sleep but were constant during the daytime All patients presented with motor stereotypies ranging from mouthing (six of 12 patients), pill rolling and twisting of two or three fingers (six of 12), bruxism (six of 12) and orofacial movements (five of 12), leg involvement (two of 12) and whole‐body stereotypy (one of 12), and tremor and myoclonus (four of 12). The myoclonus was not of epileptiform origin It was suggested that reduction of the caudate heads of thalami and presynaptic abnormalities in the nigrostriatal pathway could account for the movement abnormalities seen in patients with RTT
^26^Temudo *et al*. (2008)	Eighty‐eight patients met the revised diagnostic criteria, and, of these, 60 had an *MECP2* mutation Median age of 60 patients: 7.0 years (range: 5.0–13.5)	The cohort only included patients with *MECP2* mutations The type of mutation and its location was recorded Patients with RTT included those with the classical form (60.7%) and variant (39.3%)	Assessment scales, video observations, and genotyping	Stereotypies were noted in 95% of patients and the most common was bruxism (found in 80% of patients). Dystonia was present in 63.3% of patients, and scoliosis likely caused by truncal dystonia was noted in 71.7% of all cases. Bruxism was frequent during awake periods and did not occur at night When looking at dystonia, the study showed that focal dystonia was more common in patients with missense mutations (*n* = 26; 41.7%) when compared with those with truncating mutations (*n* = 34; 19.2%) Tremor was observed in 48.3% of all cases and were noted to be predominantly kinetic in patients with missense mutations and postural in patients with truncating mutations The study showed that movement disorders in RTT are associated with the severity and advancement of the disease. Patients with truncating mutations were shown to have a higher rate of dystonia and rigid‐akinetic syndrome It was stated that stereotypies decrease with age and become less complex and slower
^27^Downs *et al*. (2008)	Data were analyzed from 99 females with a median age of 14.1 years (range: 1.5–27.9 years)	Of the 96 individuals who had a genetic test, a confirmed *MECP2* mutation was identified in 73	Video observations alongside parent‐reported checklist	The study showed that mobility of patients decreased with age and motor scores were worse in those patients having had surgery for scoliosis Patients with the genotypes R133C, p.R294X, p.R306C, or C‐terminal deletion were said to have better mobility and complex motor skills ≥13 years and better complex motor skills <13 years of age. The development of motor skills was not impacted by behavioral hand stereotypies It was suggested that overall, motor skills—especially complex ones—were determined by the mutational profile of the patient The study also highlighted the problems associated with dyspraxia, indicating that dyspraxia is hindered by poor muscle tone and becomes more prominent when complex tasks such as transitions are performed
^28^Temudo *et al*. (2007)	Data were collected from 83 patients with RTT, of which 53 were *MECP2* mutation positive (group 1) and 30 mutation negative (group 2) Mean age of 83 patients: 10.0 years (range: 1 to 31 years)	Among the cases, 60.2% had classical RTT and 39.8% had variant RTT	Video observation in *MECP2* mutation‐positive and mutation‐negative patients	Both groups had hand stereotypies that manifested at a mean age of 22.3 months for group 1 and 25.4 months for group 2. Midline hand wringing and washing‐like movements was the most common hand movement The second most common stereotypy with hand movements was bruxism The stereotypies of hair pulling, bruxism, and cervical retropulsion was more common in group 1 (mutation positive) It was also indicated that mutation‐positive patients had more varied stereotypies and these tended to reduce after 10 years of age
^29^Einspieler *et al*. (2005)	Video analysis of 22 cases	All 22 cases had classical RTT Twelve cases were mutation positive	Motor and behavior milestones using video observations during first 6 months	All cases presented with abnormal movements (100%) followed by tongue protrusion (62%), postural stiffness (58%), and tremor (28%) Other stereotypies included hand stereotypies (42%) and stereotyped body movements (15%) The authors suggested brainstem dysfunction during neurodevelopment
^30^Wales *et al*. (2004)	Eight patients with RTT aged between 13 and 17 years	All study participants had a clinically confirmed diagnosis of classical RTT	Video observations of stereotypic hand movements	Stereotypic hand movements were found to occur in the majority of individuals The study showed that environmental modifications had limited impact on the behaviors of hand stereotypies
^31^Umansky and Watson (1998)	Nine females with RTT aged between 3 and 15 years	All patients were in the postregression phase and without speech All patients could walk, albeit with impediments None of the patients were taking AEDs	Video observations was used to define the stereotypies	The findings from the study indicated that eye movements can influence the onset of stereotypic characteristics in patients with RTT and provide further understanding into the disease post‐regression
^32^FitzGerald *et al*. (1990)	Thirty‐two patients aged between 30 months and 28 years	Forty‐one patients were seen and data were available for 32 patients Patients also met the diagnostic criteria for RTT	Video observations and MBA	The most frequent motor abnormalities noted in this cohort were stereotypies and gait abnormalities Bruxism, abnormal eye movements, and dystonia were also observed Drooling was also common (seen in 75% of patients) Young patients were noted to have more hyperkinetic movements than older patients
^33^Hirano and Taniguchi (2018)	Study information was obtained from 216 patients with RTT (age range: 3 to 53 years)	A questionnaire was sent to 1016 special needs education schools and 204 facilities in Japan	The questionnaire comprised 17 headings to assess hand stereotypies and purposeful hand behaviors	The questionnaire study showed that emotions such as displeasure (63.8%) or pleasure (48.5%) were the main factors that led to increased stereotypical hand movements Factors that decreased stereotypies were somnolence (43.5%), pleasure (30%), concentration (29.4%), and food (24.1%) It was suggested that factors that reduce behavioral stereotypies could be useful to reduce the incidence of secondary disabilities such as skin issues and joint contractures
^34^Chin Wong *et al*. (2017)	A total of 58 patients (55 females and three males) were used for analyses Typical individuals with RTT (*n* = 43) had a mean age of 15.8 ± 8.2 years and atypical cases (*n* = 15) had a mean age of 14 ± 10.4 years	Cross‐sectional study Patients were diagnosed by two neurologists based on diagnostic criteria and genetic information	Questionnaire evaluation	Of the abnormal movements, stereotypies were present in all cases—both typical and atypical (100%)—as was tremor (69%), dystonia (63.8%) agitation (62.1%), and self‐injuries (37.9%) Of the stereotypies in the 58 cases, bruxism was the most common (63.8%), followed by shifting weight from one leg to the other (63.8%), while wringing was the most common (58.6%) hand stereotypy Scoliosis caused by truncal dystonia occurred in 72.4% of cases and increased with age There were differences in the typical and atypical RTT groups. In typical RTT, shifting weight from one leg to the other (58.1%), hand wringing (58.1%), and bruxism (53.5%) were the most common stereotypies. In atypical RTT, the most common stereotypies were bruxism (93.3%), lip protrusion (93.3%), and shifting weight from one leg to the other (80%) It was suggested that basal ganglia together with cortico‐basal ganglia‐thalamo‐cortical loop involvement may play a role in the evolution of abnormal movements in patients with RTT
^35^Fehr *et al*. (2010)	The study comprised 909 cases (InterRett *n* = 570 and ARSD *n* = 339) Mean age: 28.6 years (range: 14.1–44.0 years)	Cases were obtained from InterRett and the ARSD All cases either had an *MECP2* mutation or met the diagnosis criteria according to Hagberg *et al*. (2002)^‡^. An *MECP2* mutation was found in 85.3% of cases	Clinical information based on questionnaire data	Stereotypies were found in the majority (95.1%) of cases at a mean age of 27.4 months There was also an earlier diagnosis in those patients who developed stereotypies when compared with those who did not Those patients with the p.R255X and p.R168X mutations were diagnosed at a younger age (43.7 and 43.5 months, respectively)
^36^Cianfaglione *et al*. (2015)	The study consisted of 91 females with RTT Mean age: 20.5 years (range: 4 to 47 years)	Study participants were recruited from the British Isles Rett Syndrome Survey Of the 91 females, 69 were diagnosed as having classical RTT, 19 with atypical RTT, and 3 with another *MECP2* disease	Questionnaire packs (GDQ, health questionnaire NCCPC‐R, severity scores, VABS)	The study showed that patients with truncating mutations or large deletions had greater severity Patients diagnosed with classical RTT had a higher degree of health‐related problems when compared with those with atypical RTT Stereotypies were reported in 90 of 91 (98.9%) cases and bruxism in 57.1% Frequent comorbidities in the 91 cases were epilepsy, weight, gastrointestinal, and bowel issues
^37^Stasolla and Caffò (2013)	Case study of two girls (12 and 17 years of age) with RTT	Patients had a diagnosis at 24 and 18 months of age	Microswitch‐based method using touch and optic sensors	The study showed that a microswitch‐based program could assist in managing adaptive behaviors in these individuals Improving environmental stimulation can be a positive way to manage self‐stimulation such as stereotypic behaviors, and it was suggested that this could also lower caregiver burden There was a positive impact on participants’ mood during the intervention phase; however, environmental stimuli need to be adapted accordingly to avoid sensory overload
^38^Hanks (1990)	Twenty‐three patients aged between 2 and 21 years	Confirmation of RTT in the 23 individuals was based on diagnostic criteria	Retrospective chart review of clinical data	In this retrospective chart review, there was no association between age of onset and severity of motor impairment All patients were noted to have been hypotonic during the first year postbirth Ataxia was noted in 73% of cases and 47% were ambulatory
^39^Cass *et al*. (2003)	The study cohort comprised 87 females with RTT with an age range from 2 years 1 month to 44 years 10 months	Of the 87 patients, 76 met the criteria for classical RTT and 11 met the criteria for variant or atypical RTT	Assessment schedule to assess oromotor function, feeding issues, growth, and breathing Clinical assessments were also based on patients’ needs and related questions the parents and caregivers had answered	The findings showed that there is poor growth, together with joint problems and scoliosis, that persists into adulthood Hypotonia was present in about half of the children older than 5 years but diminished after hypertonia and rigidity became established Hand stereotypies were described in 79 to 84 patients. Mouthing (45.1%) was the most common and plucking movements the least common (14.6%). Different ranges of hand use were seen among all ages In this series of patients, there was minimal change in mobility across the lifespan, and it was noted that half of the adults were mobile
^40^Jan *et al*. (2021)	Seventeen females with RTT aged 15.30 ± 8.1 years Twenty‐six healthy controls (age‐ and sex‐matched) 16.21 ± 7.9 years	All patients with RTT had confirmed *MECP2* mutations, and diagnosis was confirmed using the essential diagnostic criteria according to Neul *et al*. (2010)^†^ Because of poor imaging quality, one patient with RTT and two healthy controls were excluded from the analyses	Susceptibility‐weighted imaging methods RTT assessment scales for behavioral measurements and the Unified Dystonia Rating and Fahn–Marsden scales for dystonia assessment	The study showed that iron accumulation in brain regions was associated with the severity of dystonia in patients with RTT Dystonia assessment scales indicated that the severity was more pronounced in patients older than 10 years It was suggested that the increased iron deposition in dopaminergic networks and gray matter could account for the age‐related changes in the severity of dystonia
^41^Saikusa *et al*. (2020)	One hundred females aged between 1 and 43 years (mean ± SD: 14.5 ± 11.2 years)	Diagnosis was based on genetic and clinical diagnostic criteria Of the 100 patients, 86 had typical RTT and 14 had atypical RTT	Clinical review of information from the Japanese RTT database Genetic testing including whole exome sequencing was performed in some patients	The study showed that walking in all age groups was associated with the ability to form meaningful words In particular, the acquisition of words was associated with ambulatory ability after 10 years of age The authors concluded that patients who can walk can be predicted to form meaningful words
^43^Lai *et al*. (2021)	Database study Age ranges were 7 to 12 years (15.3%), 13 to 19 years (40.7%), and 20 years and older (44.0%)	Individuals had a confirmed *MECP2* mutation Variables that were evaluated were demographic factors, *MECP2* mutation profile, dental problems (bruxism, mouthing, and drooling), dental care, and oral health services	Review of data from InterRett including oral health variables, alongside mobility, frequency of seizures, gastric reflux, and sleep disturbances	The response rate was 93.1% (216 families of 232 that responded) The study showed that patients with bruxism were more inclined to access dental services and those who were tube‐fed had less dental experience Maternal education was suggested to be a driver for increased focus to access dental services Some patients were also said to cope with more invasive procedures such as extractions without requiring sedation
^44^Lai *et al*. (2018)	Database study of 242 females with RTT analyzed Age ranges were 6 to 20 years and older	All patients had a confirmed *MECP2* mutation	Retrospective review of longitudinal data collected from the ARSD	The study indicated that those with the most severe genotypes had worse oral health–related outcomes When adjusting for mutation, by about 3 years of age more than half of patients will have bruxism (58.74%); however, the predictive risk seems to decline with age, with 38.46% at 12 years and 16.49% at 30 years
^45^Abraham *et al*. (2015)	Twenty‐three females with a mean age of 1.7 to 5.8 years	All 23 females had a diagnosis of classical RTT and a positive test for *MECP2* mutation Stages of the disorder were assessed by a pediatric neurologist	Dysphagia was assessed using videofluoroscopic methods	Oral motility was affected by dystonic and dyskinetic movements. These movements occurred with oral apraxia during ingestion in 78% of patients There were also noticeable abnormalities in this group such as tongue retroflexion and altered (rocking and rolling) lingual patterns
^46^Cuddapah *et al*. (2014)	Participants with RTT (1052) from the RTT Natural History Study A total of 815 of 1052 patients had typical RTT, with an average age of 9.9 ± 8.9 years at enrollment A total of 148 of 1052 patients had atypical RTT with an average age of 9.1 ± 7.9 years at enrollment	Of the 1052 study participants, 963 met the diagnostic clinical criteria for either typical or atypical RTT	The clinical severity score was used to assess severity of study participants	The study findings showed that the clinical severity increases with age Ambulation, hand function, and onset of stereotypies were also associated with disease severity; however, regardless of the initial severity, the progression of RTT becomes worse with age It was indicated that X‐chromosome inactivation does not fully account for the clinical severity seen in patients
^47^Psoni *et al*. (2012)	The study included 281 patients aged between 13 months and 27 years Group 1 consisted of patients with RTT (*n* = 88) and group 2 consisted of patients with intellectual disability (*n* = 193)	Group 1 consisted of 88 children with RTT (classical RTT *n* = 40 [*MECP2* positive: *n* = 28/*MECP2* negative: *n* = 12]; variant RTT: *n* = 34 and males: *n* = 14) Diagnosis of classic or variant RTT was based on revised clinical criteria	Exon analysis: point mutations or small intraexonic deletions	Among the patients with classical RTT, there was a higher frequency of spasticity‐dystonia (46.4%) and tremor‐ataxia (64.3%) in patients who were mutation positive compared with those who were mutation negative (spasticity‐dystonia [16.7%]; tremor‐ataxia [33.3%]) The study showed that female patients who were *MECP2* positive had more difficulties in walking, muscle tone, tremor, and ataxia
^48^Bebbington *et al*. (2010)	The study consisted of 832 patients with age ranging from 8 months to 49 years Patients with C‐terminal deletions had a median age of 10 years (range: 1.3 to 43.5 years), while for patients with other *MECP2* mutations the median age was 9.3 years (range: 8 months to 49.4 years)	Of the study cohort, 79 had a deletion in the C‐terminal region and 753 had different *MECP2* mutations	The cohort was obtained from InterRett and ASRD Three severity scales were used to evaluate phenotypes	The findings from the study showed that there was a lower severity in patients with C‐terminal deletions when compared with patients without C‐terminal deletions Cases with C‐terminal deletions were also noted as having normal head circumference and weight, and the onset of stereotypes tended to be later Patients with C‐terminal deletions were also more likely to have learned to walk earlier than those with other *MECP2* mutations
^49^Monrós *et al*. (2001)	Forty‐six females and one male patient	Patients were diagnosed according to RTT diagnostic criteria	Sequencing of the *MECP2* gene	The study showed that truncating mutations were associated with greater disease severity than patients with missense mutations who had a milder course of the disease Significant differences were also noted in sitting unsupported, ambulation, and stereotypies between patients with missense and truncating mutations
^50^Bebbington *et al*. (2012)	The study cohort comprised 974 patients, with a mean age of 11.53 years (range: 1 year 4 months to 49 years) The mean age of patients with a large deletion was 9.14 years	Patients were classified per clinical criteria according to Hagberg *et al*. (2002)^‡^ Of the 974 individuals, 51 had a large deletion of *MECP2*	Data were obtained from patients recruited from InterRett and ARSD Regression and survival analysis was used to assess clinical severity	When compared with other mutations, those patients with a large deletion in *MECP2* were more severely affected and were clinically characterized by being less able to walk, less likely to have learned to walk, and having a more severe form of gross motor dysfunction Patients with large deletions also developed an earlier onset of hand stereotypies, epilepsy, and scoliosis The authors suggested that a less functional MECP2 protein could account for the clinical severity observed in muscle and motor tone phenotypes
^51^Drobnyk *et al*. (2019)	Five study participants with an age range between 3.1 and 9.1 years	The patients had RTT (classical and atypical) and RTT‐related disorders (*CDKL5*)	The study methodology utilized an interrupted time series design to assess the effects of Ayres Sensory Integration on functional reaching	Preliminary findings showed that sensory integration might offer small improvements in functional hand grasping in children with RTT Motor control appeared to increase slightly after two months of intervention and could be down to neuroplastic changes responding to environmental stimuli
^52^Stasolla *et al*. (2014)	Case series of three female patients with RTT at ages 8.4, 9.2, and 10.5 years	All females had a diagnosis of RTT at 16, 20, and 18 months, respectively	PECS and VOCA interventions	During the interventions period, the study findings noted a reduction in stereotypic behaviors in the three patients Fostering constructive engagement and promoting environmental outcomes was shown to have a positive effect on the management of stereotypic behaviors in these patients
^57^Bashina *et al*. (2002)	Fifty females with RTT aged between 12 months and 14 years	Patients had classical RTT (predominantly at stages II and III)	Clinical observations including EEG data Speech and motor disturbances were followed between 2 and 5 years	The association between the severity of motor and speech functions and neurophysiological data study points towards a dysregulation of cortical structures As the disorder progresses further subcortical, cerebellar, and spinal cord involvement can result in ataxia, tremor, and losing the ability to walk
^58^Lane *et al*. (2011)	Cross‐sectional and longitudinal (2‐year) QoL analyses of 260 individuals with classical RTT Mean age: 10 years	Data were obtained from the RTT Natural History Study	Clinical severity of patients was assessed using CSS and MBA scales	The sample showed that individuals with worse clinical status seemed to have better psychosocial functioning, i.e. patients with worse motor function together with an earlier onset of stereotypies accounted for higher QoL scores There were no changes in QoL scores and clinical severity over the 2‐year duration
^59^Gika *et al*. (2010)	Case presentation of three patients with RTT aged 9, 13, and 20 years	Patients had ALTEs and patient #3 died before trihexyphenidyl could be used	Review of case histories	ALTEs were nonepileptic in nature In two of the patients (patient 1 and 2), use of trihexyphenidyl reduced ALTEs caused by dystonia

^†^ Neul JL, Kaufmann WE, Glaze DG, Christodoulou J, Clarke AJ, *et al*. RettSearch Consortium. Rett syndrome: revised diagnostic criteria and nomenclature. *Ann Neurol*. 2010; 68(6): 944–950.

^‡^ Hagberg B, Hanefeld F, Percy A, Skjeldal O. An update on clinically applicable diagnostic criteria in Rett syndrome. Comments to Rett Syndrome Clinical Criteria Consensus Panel Satellite to European Pediatric Neurology Society Meeting, Baden Baden, Germany, 11 September 2001. *Eur J Paediatr Neurol*. 2002; 6(5):293–297.

AED, antiepileptic drug; ALTE, acute life‐threatening episode; ARSD, Australian Rett Syndrome Database; ASD, autistic spectrum disorder; CDKL5, cyclin‐dependent kinase‐like 5; CSS, Clinical Severity Scale; DSM‐III‐R, Diagnostic and Statistical Manual of Mental Disorders–Third Edition Revised; EEG, electroencephalography; GDQ, Gastro‐esophageal Distress Questionnaire; InterRett, International Rett Syndrome Phenotype Database; MBA, Motor‐Behavioral Assessment; *MECP2*, methyl‐CpG binding protein 2; NCCPC‐R, Non‐communicating Children's Pain Checklist – Revised; PECS, Picture Exchange Communication System; QoL, quality of life; RCT, randomized controlled trial; RTT, Rett syndrome; VABS, Vineland Adaptive Behavior Scale; VOCA, voice output communication aid; WeeFIM, Functional Independence Measure for Children.

### Characteristics of the eligible articles

As presented in Table 1, the 43 studies captured information across the movement disorder ecosystem in patients with RTT. About 42% of the studies used video observations to assess movement disorders.^12^, ^16^, ^17^, ^18^, ^19^, ^20^, ^21^, ^22^, ^23^, ^24^, ^25^, ^26^, ^27^, ^28^, ^29^, ^30^, ^31^, ^32^ Others used questionnaires as the primary assessment method^11^, ^33^, ^34^, ^35^, ^36^ or as an adjunct with other methods.^18^ The sample was also diverse, from an analysis of 1074 patients with RTT from the RTT Natural History Study,^10^ to a study exploring a few cases.^37^ The data obtained allowed a thematic analysis to be performed and are presented in the next section.

### A thematic analysis of the studies

**Fig. 1:**
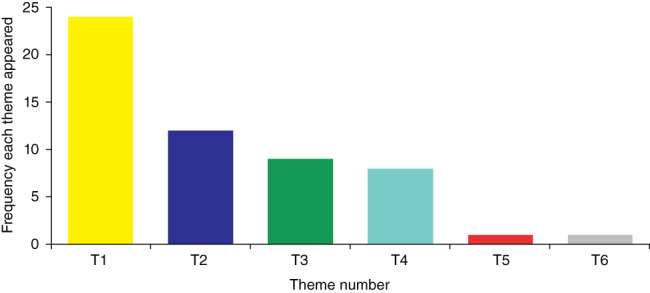
Frequency of six identified themes. Key: 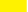, Theme 1: Clinical features of abnormal movement behaviors; 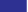, Theme 2: Mutational profile and its impact on movement disorders; 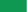, Theme 3: Symptoms and stressors that impact on movement disorders; 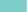, Theme 4: Possible underlying neurobiological mechanisms; 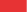, Theme 5: Quality of life and movement disorders; 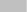, Theme 6: Treatment of movement disorders.

When extracting data from the 43 eligible articles, six themes emerged from the thematic analysis. The themes encompassed different aspects of the movement disorder ecosystem in patients with RTT and, where appropriate, subgroups of movement disorders within the themes have been indicated. Some of the themes, such as those related to underlying neurobiological mechanisms, QoL, and treatment of movement disorders, had the lowest frequency and underscores the importance of much‐needed work in these specific areas. Nonetheless, ‘Clinical features of abnormal movement behaviors’ emerged as the most prominent theme followed by ‘Mutational profile and its impact on movement disorders’. The relevance of these themes is described below and their frequency is shown in Fig. 1.

#### Theme 1: Clinical features of abnormal movement behaviors

##### Dystonia

A high proportion of patients with RTT present with dystonia,^26^ and scoliosis caused by truncal dystonia was also shown to increase with age.^34^ From a developmental perspective, hypotonia is noted to be present in children with RTT during the first year after birth^38^ and present in about 50% of children younger than 5 years but seems to get less as the child becomes older when hypertonia and dystonic type rigidity become established.^39^ This is clinically interesting because another study has shown that as the disorder progresses, features such as dyspraxia are hindered by poor muscle tone and become more prominent when complex tasks are required to be performed.^27^ In another study, the clinical severity of dystonia was associated with iron accumulation in brain regions of patients with RTT.^40^

##### Parkinsonian‐like features/tremors and ataxia

Parkinsonian rigidity is prevalent in patients with RTT, accounting for about 84% in one study.^15^ It tends to have an early onset (3 years of age) and increases with age. The rigidity usually starts with the ankle, and nonambulatory patients are more severely affected. In patients who were ambulatory, freezing of gait appeared to be the most characteristic movement behavior.^16^ Patients who could walk were also more inclined to have the ability to form meaningful word acquisitions.^41^ Interestingly, video and treadmill walking observations were also helpful in assessing ataxic gait. These features would be useful in discriminating between traditional ‘Parkinsonian’ signs observed from freezing behavior to those of cerebellar origin such as ataxia.

##### Stereotypies

Clinical severity is worse in patients with decreased hand function,^10^ and increases in bradykinesia and hypertonia are thought to have a role in hand functioning. As suggested by others,^17^ hand stereotypies have a heterogenous phenotype. Hand mouthing and clapping/tapping were more common than hand wringing/washing,^10^ but hand wringing was the most common hand stereotypy in another study.^34^ Moreover, some stereotypies such as hand^25^ and bruxism^26^ disappear at night. One clinical feature that was highlighted was unilateral hand movements associated with centrotemporal spikes on the electroencephalogram (EEG). These were suggested not to be epileptic but rather somatosensory or motor potential spikes originating from different brain regions.^20^ Abnormal EEGs in RTT evoked by hand clapping have also been reported elsewhere.^42^ These cases are useful because they show that although abnormal movements may evoke centrotemporal spikes on the EEG, they do not necessarily indicate that this is caused by epilepsy and highlights the importance of routine monitoring of movement disorders in this patient group.

Other stereotypies are also prevalent in patients with RTT, and these too can emerge differently, such as bruxism^26^, ^28^ and shifting of the weight.^34^ Individuals with bruxism were also more inclined to access dental services^43^; however, the risk of bruxism appears to decline with age.^44^ Oral motility was also affected by dystonic and dyskinetic movements.^45^ Other evidence suggests that while stereotypies and hand functioning remained stable over time, musculoskeletal problems became worse, and this feature continues into adulthood.^11^ Regardless of the initial severity, ambulation, hand functioning, and onset of stereotypies is suggested to get worse as the disorder progresses.^46^

##### Other features

Another study has also highlighted tongue protrusion (62%) and postural stiffness (58%) in patients,^29^ while recognizing patterns in eye movements could assist in predicting the onset of stereotypic behaviors.^31^

There is overlap between this theme (theme 1) and the mutational profile of patients and how this profile affects movement disorders (theme 2). Frequent comorbidities appear to be epilepsy, weight, gastrointestinal, and bowel movements in those with truncating mutations or large deletions.^36^ The mutational profile of patients with RTT and its impact on movement disorders is described in theme 2.

#### Theme 2: Mutational profile and its impact on movement disorders

This theme provided an enriched data set that allows deeper exploration of the impact of the genotype–phenotype relationship on movement disorders in RTT.

##### Dystonia

When evaluating gross motor disturbances, focal dystonia is more common in patients with missense mutations than with truncating mutations. In contrast, individuals with truncating mutations tended to have a higher frequency of generalized dystonia. Additionally, the appearance of tremor can also be different. Overall, the frequency of dystonia and rigid‐akinetic syndrome was also higher in patients with truncating mutations.^26^

##### Parkinsonian‐like features/tremors and ataxia

When assessing Parkinsonian rigidity scores in RTT, there was no difference between patients with truncating and missense mutations^15^; however, patients who were less able to walk presented with worse scores on the rigidity scale. Kinetic tremors were more common with missense mutations when compared with truncating mutations where postural tremors were more abundant.^26^ In the Greek sample of patients with classical RTT, the frequency of spasticity‐dystonia and tremor‐ataxia was higher in individuals who were mutation positive.^47^

##### Stereotypies

One study has suggested no association between hand stereotypies and genotype,^12^ while other evidence has indicated that patients with the p.R168X mutation had the poorest hand function.^23^ The number of stereotypies in individuals with missense or truncating mutations were similar; however, rigidity and dystonia were more common in individuals with truncating mutations.^22^ Individuals with R133C, p.R294X, p.R306C mutations, or a C‐terminal deletion were also said to have better mobility and complex motor skills when 13 years or older and better complex motor skills <13 years of age.^27^ Hair pulling, bruxism, and cervical retropulsion were more common in mutation‐positive patients.^28^ When individuals with typical and atypical RTT were compared, shifting weight on one leg, followed by handwringing and bruxism, were the most common stereotypies in individuals with typical RTT. On the contrary, bruxism and lip protrusion were the most common in patients with atypical RTT.^34^

Clinical information from the International Rett Syndrome Phenotype Database (InterRett) and the Australian Rett Syndrome Database showed that patients with R255X and p.R168X mutations tended to be diagnosed at a younger age,^35^ with developmental regression starting sooner. This is relevant because the study also indicated that an older age of diagnosis, especially in individuals with the p.R133C or p.R294X mutations, was linked to delayed loss of speech and hand functioning.^35^ Individuals with C‐terminal deletions were deemed to be less clinically severe and more likely to have learned to walk,^48^ while truncating mutations were associated with worse clinical outcomes.^49^

##### Other features

In a survey of patients from the British Isles RTT database, individuals with truncating mutations or large deletions had greater clinical severity.^36^ These patients can also be clinically characterized as not being able to walk, not having actually learned to walk, and having the worst form of gross motor dysfunction.^50^

#### Theme 3: Symptoms and stressors that impact on movement disorders

This theme underscores the influence of symptoms and stressors on movement disorders. The environment is important in managing negative behavior associated with self‐injury in RTT.^18^ High‐stress conditions can also lead to visible signs of displeasure in the faces and the vocalization of patients with RTT^19^ and could have implications on how movement behaviors can be affected by these signs. Evidence further suggests that environmental and sensory influences might have an important role in reducing the incidence of secondary disabilities associated with abnormal movements in RTT.^33^ In this questionnaire‐based study, factors that led to a decrease in stereotypies were somnolence, pleasure, concentration, and food. These factors were said to lower the incidence of secondary skin issues and joint contractures associated with stereotypies. This premise was also supported by others who have suggested that constructive engagement through promotion of different environmental and sensory factors might have a positive influence on managing stereotypic behavior in patients with RTT.^23^, ^24^, ^37^, ^51^, ^52^ However, there are data to also suggest that environmental modifications have limited impact on the behavior of hand stereotypies.^30^

The neurobiological relationships between stressors and movement disorders remain unclear; however, areas within the basal ganglia and the medial prefrontal cortex are involved in regulating motor outputs based on emotional states.^53^, ^54^ Stress can also have a profound impact on dopaminergic networks, and, alongside the hypothalamic–pituitary–adrenal axis, are vital for responding to rapidly changing environmental cues.^55^, ^56^ In RTT, these pathways could be negatively impacted by stress, anxiety, or depression, resulting in a deterioration of fine and gross motor function.

#### Theme 4: Possible underlying neurobiological mechanisms

The neurobiological mechanisms of movement disorders was another theme that emerged from eight studies. In RTT, the underlying mechanisms appear to be diverse.

##### Dystonia

Increased iron accumulation in dopaminergic networks and gray matter was suggested to be correlated with the severity of dystonia in patients with RTT.^40^

##### Parkinsonian‐like features/tremors and ataxia

Earlier studies have suggested the involvement of neural mechanisms associated with the reduction of caudate heads, thalami, and presynaptic abnormalities within nigrostriatal pathways.^25^ As the disorder progresses, deterioration of subcortical, cerebellar, and spinal cord networks causes ataxia and tremor and impedes the ability to walk.^57^

##### Stereotypies

The phenotype of movement disorders might also present with different neural mechanisms. In patients with RTT, stereotypic behaviors are not observed during freezing of gait, which could suggest that the neural mechanisms underlying these processes could be independent from one another.^16^

##### Other features

Some studies have suggested that the cortico‐basal ganglia‐thalamo‐cortical (CBGTC) loop is implicated in the evolution of movement disorders in RTT.^21^, ^34^ Brainstem and cerebellar structures have also been linked when explaining the phenotype of movement disorders.^22^, ^29^

#### Theme 5: QoL and movement disorders

This theme was relevant in the context of this evidence synthesis because it explored how psychosocial aspects of movement disorders can affect QoL. In a longitudinal 2‐year follow‐up of 260 patients enrolled in the RTT Natural History Study, patients with worse motor functions and an earlier onset of stereotypies had higher QoL scores on psychosocial functioning.^58^ The authors reasoned that patients with more severe motor abnormalities were less likely to have negative behaviors (aggression and self‐injurious behavior) that would have otherwise impacted negatively on their psychosocial QoL. This finding is relevant because it suggests that when assessing severe clinical impairments in RTT under the umbrella of movement disorders, the changes in QoL are not uniform and the outcomes can be variable.

#### Theme 6: Treatment of movement disorders

This theme emerged from one study. In a small case series of three patients with RTT (age range: 9–20 years), acute life‐threatening episodes caused by dystonic movements could be managed in two patients using trihexyphenidyl.^59^

Movement disorders can emerge with different clinical phenotypes spanning across dystonia, Parkinsonism, bruxism, spasticity, tremor, and ataxia. While there is useful information on clinical practices for managing pediatric movement disorders,^60^ improved diagnostics for dystonia,^61^ and guidance on the management of stereotypies,^62^, ^63^ there is little information on the treatment of movement disorders. In the following section, general treatments for movement disorders and their implications for patients with RTT will be discussed.

## Discussion

The current evidence synthesis evaluated a range of clinical and neurobiological features across the spectrum of movement disorders in patients with RTT. It aimed to identify themes and suggested how the information in these themes could be extrapolated and used by clinicians and other healthcare professionals to inform the wider RTT community. The main findings identified in this study were: (i) further knowledge and learning of the clinical features of movement impairments in patients with RTT; (ii) a synthesis of information regarding genotype–phenotype relationships of movement disorders in RTT; (iii) symptoms and stressors that impact on movement disorders; (iv) deeper insight into the possible underlying neurobiological mechanisms; (v) how the QoL of patients with RTT, especially the psychosocial aspect, can be affected by movement disorders; and (vi) treatment implications for managing movement disorders in patients with RTT.

The merit of each of these themes and the associated clinical considerations are described in the next section.

### Clinical features of abnormal movement behaviors

Movement disorders in RTT are wide‐ranging. The impairments in gross and fine motor functioning also overlap with other organ systems, making their treatment challenging. The clinical features that emerged in theme 1 can supplement our understanding of movement disorders in RTT across the broader clinical ecosystem. Our review suggests that the developmental trajectory of movement disorders as the disorder advances is variable and not well defined (Table 2). This is important because in RTT, other symptoms such as cardiorespiratory fatigue and EBAD are likely to lead to worsening motor function and underscores the need for ongoing clinical surveillance of movement disorders in this patient group.

**Table 2:** Developmental trajectory of movement disorders in patients with RTT

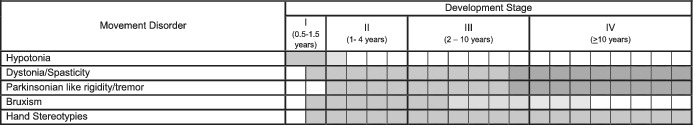

Stage I (period of developmental stagnation) between 6 to 18 months of age. During the initial period there is hypotonia and this lessens after rigidity becomes established. Stage II (developmental regression phase) between 12 to 48 months. Parkinsonian‐like rigidity is common. It can appear early (3 years of age) and frequency increases with age. Nonambulatory patients are more severely affected. In the later parts of stage I and beginning of stage II, patients can present with bruxism. When adjusting for mutation type, bruxism declines with age. For the majority of patients, hand stereotypies appear during this stage, but hand stereotypes may also appear before regression in some patients. Hand function declines over time; however, the frequency of hand stereotypies remain stable and high across the lifespan. Stage III (pseudostationary stage) around ages 2 to 10 years. Dystonia and Parkinsonian‐like rigidity persists across the lifespan of the disorder and is usually stable. The severity and trajectory of dystonia, spasticity, and Parkinsonian‐like movement disorders are probably dependent on the genotype, especially in patients with truncating mutations or large deletions who have worse clinical symptoms and outcomes. Stage IV (motor regression) about 10 years of age. Patients enter into motor regression, which is primarily associated with a decline in gross motor function. RTT, Rett syndrome.

#### Dystonia

While we have previously indicated that proactive management of dystonia would be useful for managing autonomic dysregulation and hence EBAD in patients with RTT,^64^ dystonia is relatively understudied in RTT. Dystonia is often misdiagnosed in other neurodevelopmental disorders^65^, ^66^ and an inaccurate diagnosis of spasticity or dystonia might lead to delays in optimizing surgical strategies. In patients with RTT, scoliosis caused by truncal dystonia can increase with age^34^ and can exacerbate the gross motor decline if it goes unmanaged. Further data from the RTT Natural History Study show that patients who were more likely to develop scoliosis were less able to have functional use of their hands.^67^

The diagnosis of dystonia in RTT can be difficult as it is often associated with other movement disorders. The nonmotor components of primary dystonia, such as sensory and neuropsychiatric abnormalities, prevalent in RTT, also need to be carefully considered^68^ alongside Sandifer syndrome, which is commonly mistaken for dystonia. Given the gastrointestinal dysfunction in RTT, close attention regarding Sandifer syndrome is also needed to avoid misdiagnosis and wrong treatment. Clinical signs and symptoms of paroxysmal autonomic instability with dystonia (PAID) syndrome should also be considered.^69^ PAID may present in cases where there is already an underlying autonomic dysregulation. Moving forward, the identification of clinical features and severity of dystonia in RTT could be improved by adopting the recommendations of dystonia rating scales.^70^

#### Parkinsonian‐like features/tremors and ataxia

Freezing and ataxic gait in patients could reveal distinct neurological mechanisms. In patients with RTT, Parkinsonian‐like rigidity is frequent and usually appears early on in development and tends to increase with age.^15^ This may further contribute to the gross motor decline.

#### Stereotypic movements

Specific movement behaviors such as hand stereotypies can be adversely affected by bradykinesia and hypertonia.^10^ Bruxism is a common stereotypic behavior in patients with RTT and also appears to be the most common oral issue.^71^ Other evidence has shown a relationship between bruxism and gastroesophageal reflux disease^72^ and anxiety.^73^ Because gastrointestinal issues and anxiety can worsen EBAD in patients, it would be prudent to monitor EBAD in RTT to facilitate the management of bruxism.

Nighttime bruxism is less frequent in patients^74^ and, in general, the stereotypies associated with hand functioning and bruxism in patients with RTT seem to disappear at night. However, the mechanism behind this is unknown but is likely to implicate independent neural mechanisms. In RTT, autonomic dysregulation is present during wakefulness and also at night,^75^, ^76^ suggesting that the dampening down of fine motor deficits (hand stereotypies and bruxism) during sleep involves neural substrates not influenced by autonomic dysregulation, or could be more resilient to them. Further investigation would be needed to test this hypothesis.

### Mutational profile and its impact on movement disorders

The second most frequently occurring theme was associated with how the mutational landscape in RTT impacts the clinical severity of movement impairments. This showed that: (i) the phenotype of movement disorders is different between typical and atypical RTT; (ii) overall, those patients with truncating mutations or large deletions had worse clinical symptoms and outcomes; and (iii) C‐terminal deletions were less severe in these patients. As many specialist clinicians are involved in the care of patients with RTT, they might not be aware of the diversity of relationships between different movement disorders and the mutational profile in RTT. This could lead to delays in diagnosis or adoption of a ‘wait‐and‐see approach’ as suggested by others.^35^ Our review has provided a valuable resource of information regarding mutation and clinical impact by adding to the evidence base. Despite not following a predicted clinical trajectory, when it comes to the management of movement disorders in RTT, this information would be useful for assisting clinicians in better counseling families.

#### Dystonia

Dystonia appears to be common in individuals with truncating mutations; however, as dystonia may show different distribution it can emerge sporadically during the lifespan. Age‐related changes of dystonia in RTT are difficult to predict even if a clear mutational profile has been established.

#### Parkinsonian‐like features/tremors and ataxia

The frequency of Parkinsonian‐like rigidity does not differ between individuals with truncating and missense mutations.^15^ Some other patterns also emerge, such as R294X mutations presenting with hyperactive behavior and those with the T158M mutation having an ataxic rigid phenotype.^22^

#### Stereotypic movements

Individuals with severe genotypes have worse oral health–related outcomes and by the age of 3 years >50% will have bruxism; however, when adjusting for mutation, the predictive risk of bruxism decreases with age.^44^ The genotype–phenotype relationship of hand stereotypies is less clear.^10^, ^12^, ^27^, ^77^ Fine motor disturbances such as those caused by hand stereotypies can also appear in patients who are mutation‐negative for methyl‐CpG binding protein 2 (MECP2), suggesting that mutations in *MECP2* are not entirely responsible for driving the neurobiological impairment seen in hand stereotypies.^10^

It would be helpful to develop objective measures to identify patterns of stereotypies among different mutations. Machine learning could be a useful foil alongside wearable sensors to track movements in patients with RTT and help in the classification of repetitive movement patterns. In the context of this evidence synthesis, the information regarding the mutational profile and movement disorders is useful because it enriches the preexisting evidence base.

### Symptoms and stressors that impact movement disorders

Sensory factors and stressors influence movement disorders in RTT. In some instances, sensory influences might help to reduce the incidence of secondary disabilities^33^; however, in RTT, the symptoms of EBAD can worsen because of acoustic sensitivity, pain, and other coexisting symptoms and stressors.^2^ The input of the sensory pathway could be altered in patients with RTT and this could make individuals more vulnerable to altered somatosensory processing especially in patients who are most at risk, i.e. less ambulatory or confined to a wheelchair. A decrease in pain sensitivity forms part of the supportive diagnostic criteria in patients^1^; however, a decreased pain sensitivity could make this patient group more susceptible to chronic pain. This is supported by the observation that although patients with RTT do experience chronic pain, their pain expression remains unaltered,^78^ implying that the pain response could be clinically reflected by a worsening of EBAD symptoms and movement behaviors. Visual cues of EBAD symptoms such as pain may also be blunted by impaired movements^79^ and because previous reports have suggested that parents were unsure of whether their child had experienced pain in the last month,^80^ it reinforces the notion that proactive monitoring of EBAD symptoms to reduce sensory stressors are critical in RTT. Baseline heart rate variability^81^ and using electrodermal activity to monitor the impact of stressors on chronic illness in patients with RTT^82^ could be options to assist clinicians in the management of EBAD and its impact on movement disorders in this patient group.

### Possible underlying neurobiological mechanisms

As far as we are aware, our evidence synthesis, combined with thematic analysis, is the first to reveal the underlying neurobiological mechanisms among the movement disorder spectrum in patients with RTT. In RTT, a different neural mechanism can operate with regards to movement disorders, as was noted between freezing of gait and ataxic gait,^16^ and these neural systems are probably independent of the pathways associated with autonomic dysregulation. The evidence revealed that abnormalities of the basal ganglia and dysregulation of the CBGTC loop are likely to be involved. Some of the age‐related changes of movement disorders was suggested to be caused by changes in neurotransmitter density in the basal ganglion.^34^ There has also been some indication of nigrostriatal pathway involvement.^25^, ^32^, ^83^, ^84^ Animal models further demonstrate nigrostriatal deficits.^85^, ^86^ Despite these findings, the relative contribution of the loss of nigrostriatal circuits and associated movement disorders in RTT is still unknown. Previous imaging studies in patients suggest the impact of nigrostriatal pathway activity to be mild.^87^ Homovanillic acid (HVA) level is also negatively correlated with Parkinsonian‐like rigidity in patients with RTT,^15^ demonstrating a more specified dopaminergic involvement. Although lower levels of HVA could be associated with rigidity in patients with RTT, an extensive study of HVA levels in 1388 children with neurological disorders suggests that abnormalities in HVA levels is a frequent finding in different neurological conditions and, in some cases, probably not disease specific.^88^ Nonetheless, measurement of HVA levels in RTT in a larger sample population and how these levels correlate with white matter changes^88^ would be useful in furthering our understanding of the role of dopamine metabolism and movement disorders in RTT.

Studies in mice models of RTT have also suggested that motor dysfunction arises from cerebellar dysfunction^89^ and repetitive movement behaviors caused by GABAergic dysfunction^90^; however, how this relates to different patient populations and the underpinning clinical severity remains to be established. While an imaging study in females with RTT emphasizes reductions in the volume of parietal gray matter and anterior frontal lobe^91^ as the most prominent anatomical abnormalities, whether these anatomical changes cause different movement impairments in patients with RTT is unknown. Recent sensitive neuroimaging investigations in patients with RTT using a susceptibility weighted imaging approach have indicated increased iron deposition in dopaminergic, and gray matter networks of the basal ganglia are associated with the severity of dystonia.^40^ When viewed together, a better understanding of the neurological underpinning of movement disorders in patients with RTT has provided an enriched picture of the different neural circuits involved. This may help to assist future studies of brain imaging in RTT.

### QoL and movement disorders

Understanding how abnormal movement behaviors affect QoL in RTT is not straightforward. When QoL is evaluated using the Child Health Questionnaire 50 (CHQ‐PF50), patients with RTT with more severe motor impairments had better psychosocial functioning because they were less likely to engage in maladaptive behaviors such as aggression and self‐injury.^58^ In a recent observational study that assessed the QoL more broadly among patient with intellectual disability, including those with RTT, cerebral palsy, and Down syndrome, multivariate analysis showed that mobility was less influential on QoL.^92^ This study used the validated Quality of Life Inventory‐Disability (QI‐Disability) tool to assess QoL. Using different tools to assess QoL in patients with complex neurodisability makes it challenging to infer clinical patterns among studies when examining how mobility and other aspects of movement disorders in RTT affect QoL.

Even when different instruments are used to assess QoL, in RTT, the genotype–phenotype relationship can also confound interpretation. In a study of 210 patients with RTT, even though the p.Arg294 mutation was the clinically milder phenotype, patients with the p.Arg294 mutation had the poorest QoL scores overall^3^ and partly supports the previous observation regarding psychosocial summary.^58^ This finding is important because it could be possible that in those patients with the milder clinical form, the behavioral and emotional components of EBAD could exacerbate the clinical symptoms of movement problems in RTT, which could negatively affect QoL but might not be necessarily captured using existing QoL tools. Reporting of QoL domains may also differ,^92^ as do the discrimination between health‐related QoL and QoL overall. While we cannot speculate on the relationship between the clinical symptoms of EBAD and genotype on QoL, we know that the QoL of patients with RTT worsens after 12 years of age.^3^ Therefore, it would be important when planning future treatment and rehabilitation programs to longitudinally track the effect of EBAD on movement disorders using an appropriate QoL measure as the disorder progresses.

### Treatment of movement disorders

Information regarding the treatment of movement disorders is limited in patients with RTT. From the 43 manuscripts evaluated, only one presented a small case series of two patients using trihexyphenidyl to assist in the management of life‐threatening events arising from dystonic episodes.^59^ By completing our systematic review followed by a thematic analysis, we can use this information to further understand the treatment of movement disorders from a wider perspective. When considering treatment of movement disorders in patients with RTT, reduction in movement is not the primary goal. Rather, the goal of treatment should be placed on reducing impairment and achieving functional movement.

As we have previously mentioned,^64^ information can be extrapolated from other observations in the non‐RTT population to inform our current understanding. We can now apply this knowledge to movement disorders in RTT. There is little evidence to support the use of different treatments in movement disorders. Notwithstanding this limitation, different treatment options and how they can be extrapolated to the RTT population to inform current guidelines are discussed.

#### Pharmacological interventions

**Fig. 2:**
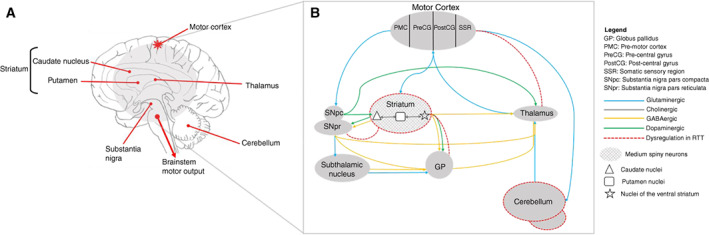
Schematic of brain regions implicated in movement disorders and associated neurochemical pathways. Panel A: Cross‐section of the human brain showing different regions likely to be involved in movement disorders. Panel B: The array of neurochemical pathways associated with movement are complex^93^ with multiple interconnecting circuits that could be dysregulated in Rett syndrome (RTT).^96^ In our simplified adaptation of network schematics,^93^, ^96^ outputs from striatal medium spiny neurons project into the substantia nigra pars reticulata (SNpr) via the striatonigral pathway or into the globus pallidus (GP) via the striatopallidal pathway. These projections modulate thalamic activity and trigger or diminish motor activity through dopaminergic or GABAergic control. Impairments in dopaminergic or GABAergic circuits may give rise to the Parkinsonian‐like movement features or repetitive behaviors in RTT.^15^, ^90^ Striatal integrity may also be dependent on functional methyl‐CpG binding protein 2.^96^ More recent evidence in mice models further suggests that motor impairments in RTT could arise from altered cerebellar architecture.^89^ Evidence also indicates glutamate receptor dysregulation in the motor cortex of postmortem brain tissue in patients with RTT^97^ and this may affect neurotransmission into the thalamus. Aspects of network dysregulation that may influence movement disorders in RTT are shown.

The brain circuitry involved in movement disorders implicates cholinergic, dopaminergic, GABAergic, glutaminergic, and other basal ganglia networks.^63^, ^73^, ^93^, ^94^, ^95^ These networks can be impaired in RTT.^89^, ^90^, ^96^, ^97^ Figure 2 shows a schematic adaptation of these networks and their potential dysregulation in RTT.

As there is little research on abnormal movement treatment in RTT, we extended our search to include management of abnormal movements in other conditions in children and young people. Baclofen, gabapentin, trihexyphenidyl, levodopa, diazepam, and clonidine are common medications for managing impaired muscle tone.^98^, ^99^

Anticholinergics such as trihexyphenidyl was shown to be useful to assist in the management of dystonia in a case study of two patients with RTT.^59^ There is little to no evidence on the effectiveness of trihexyphenidyl for treating dystonia in children with cerebral palsy.^100^

Baclofen should be used with caution as there are no evidence‐based studies on the effectiveness of using baclofen to manage dystonia in patients with RTT. The use of intrathecal baclofen in dystonia and spasticity is associated with complications that might also require further surgery.^101^ Close attention should be placed on the recognition of hypoventilation and worsening of extrapyramidal symptoms in RTT.

Benzodiazepines such as clonazepam have also been used for the management of movement disorders such as dystonia despite their efficacy not being proven. However, as we have previously indicated,^14^ the use of benzodiazepines should only be used in RTT when deemed strictly necessary. If benzodiazepines are prescribed, their use must be monitored carefully because of the high potential of worsening of autonomic function in this patient group.

α_2_‐Agonists such as clonidine have been shown to be effective in managing secondary dystonia in a cohort of patients with cerebral palsy.^102^ However, a recent meta‐analysis indicated that the level of evidence for clonidine treatment in improving dystonia in cerebral palsy was low to very low.^100^ Some data in a small number of cases suggest that clonidine might be useful for the treatment of acute akathisia.^103^, ^104^, ^105^ Clonidine must be used with caution given its multisystemic side effect profile.^64^ The known side effects of clonidine such as sedation, hypotension, and mood changes are especially relevant in RTT. There are no published studies of clonidine for managing movement disorders in RTT.

Gabapentin has been shown to reduce the severity of dystonia and improve the QoL of children in a retrospective observational study involving 69 children with refractory dystonia.^106^ Gabapentin might also be useful for treating some cases of essential tremor.^107^, ^108^, ^109^ Again, the effectiveness of gabapentin for the management of dystonia in patients with cerebral palsy has not been established.^100^, ^110^, ^111^ Gabapentin must be used with caution given its side effect profile,^64^ and it can also induce movement disorder onset.^112^, ^113^ There is no published evidence on the efficacy of gabapentin for treating abnormal tone in RTT.

Dopaminergics have also been used to manage dystonia; very low‐dose levodopa therapy is another option to treat the symptoms of motor impairments in childhood neurological disorders.^114^ However, studies in RTT are needed to explore its efficacy and side effect profile, as their use can cause nausea and constipation. Gastrointestinal abnormalities such as constipation are prevalent in patients with RTT, and, therefore, dopaminergics could potentially worsen constipation in this patient group.

Botulinum toxin might be useful in treating focal dystonia.^95^ Despite this observation, there is no clear evidence whether botulinum toxin would be useful for treating movement disorders in patients with RTT. A small pilot study of patients with RTT has suggested that botulinum toxin was helpful in treating hypersalivation and may also improve other oral functions.^115^ As the minimum dosing interval is about 12 weeks, long periods between injections might also be required on an individual patient basis.

In summary, the lack of empirical evidence, inadequate evidence of efficacy, and the potential for multisystem side effects of pharmacological agents being used currently necessitates the need for close monitoring in patients with RTT. These treatments and the implications for using them in patients with RTT are summarized in Table 3.

**Table 3:** Treatment of movement disorders – implications for the management of patients with RTT

Movement disorder	Brain circuitry involved	Treatments	Considerations for patients with RTT
Hypertonia (dystonia/spasticity)	Cholinergic, dopaminergic, GABAergic, glutaminergic	Anticholinergics, baclofen, BDZ, dopaminergics, Botulinum toxin, α_2_‐Agonist, gabapentin	*Anticholinergics* Trihexyphenidyl is used to target dystonia. The most common side effects include a reduction in concentration and memory, which might not easily be detected in patients with RTT. Peripheral side effects can include dry mouth, urinary retention, constipation, and blurred vision. This could increase the risk of urinary tract infections and worsen preexistent gut dismotility. Sudden discontinuation can precipitate a change in mental state. *Baclofen* An analogue of GABA, it targets spasticity and dystonia. Oral baclofen can cause dose‐dependent side effects, which include sedation, hypoventilation, and increased seizures, thus possibly worsening preexisting breathing problems and/or epilepsy. *Benzodiazepines* BDZs can be prescribed to target spasticity and dystonia. Their use should be avoided in patients with RTT because: (i) BDZ could induce respiratory depression for which patients with RTT already have a vulnerability; (ii) BDZ such as clonazepam can cause excess drooling, thus increasing the risk of aspiration pneumonia; and (iii) BDZ can cause paradoxical agitation in patients with neurodevelopmental disorders. *α*_*2*_ *‐Agonists* α_2_‐Agonists such as clonidine can assist in the management of secondary dystonia in other patient groups. Clonidine has the potential to increase adverse events. There are no published studies on the effectiveness of clonidine for treating movement disorders in patients with RTT. *Gabapentin* It was shown to improve muscle tone in children with refractory dystonia. In patients with RTT, gabapentin must be used with caution given the lack of empirical evidence in RTT and the risk of multisystem side effects. *Dopaminergics* Carbidopa/levodopa can assist in the management of dystonia. An increase in nausea and worsening of constipation must be carefully monitored. Longer‐term treatment of dopamine could worsen bruxism. *Botulinum toxin* Targets focal and segmental dystonia/spasticity by blocking acetylcholine release at the neuromuscular junction, thus inducing a transient muscle relaxation. Reinjection is necessary at 12–14 weeks. The injection site procedure is minimally invasive for superficial muscles but can require sedation, which has additional complications for patients with RTT.
Parkinsonian features	Cholinergic, dopaminergic, GABAergic, glutaminergic	Anticholinergics, dopaminergics, NMDA receptor antagonist	*Dopaminergics* Parkinsonian features in patients with RTT usually do not respond to levodopa or dopamine agonists, possibly because of a postsynaptic defect.
Abnormal gait (ataxic/dyspraxic)	Cerebellar networks		No medication has proved to be successful in improving gait abnormalities in patients with RTT.
Stereotypies/bruxism	Limbic parts of basal ganglia networks^†^		The efficacy of SSRIs for repetitive movements has not been demonstrated; however, when prescribed to target anxiety, there could be a secondary behavioral improvement.

^†^ There is basal ganglia involvement; however, the neuropathology of stereotypies remains incomplete and is likely to involve other branches of cholinergic and dopaminergic pathways.

BDZ, benzodiazepine; GABA, γ‐aminobutyric acid; NMDA, N‐methyl‐d‐aspartate; RTT, Rett syndrome; SSRIs, selective serotonin reuptake inhibitors.

#### Surgical and minimally invasive based options

There is some evidence that intrathecal baclofen and surgical treatments such as deep brain stimulation (DBS) of the globus pallidus pars interna are helpful in treating acquired or refractory dystonia in patients with cerebral palsy.^116^, ^117^ Another study has shown that pallidal deep brain stimulation for dystonia should be considered early on in childhood as the response becomes less effective as the dystonia becomes more established.^118^ There are other aspects of DBS surgery for the treatment of dystonia that need to be considered. Psychological support preintervention and postintervention should be explored to assess the impact of DBS on social functioning and QoL in the longer term.^119^ It is unclear whether such interventions would be feasible for patients with RTT. There is little information on the surgical management of dystonia in patients with RTT.

#### Exploratory strategies and neuroprotection

Exploratory strategies might help to reduce the progression of movement disorders in RTT. Patients with RTT with severe dystonia have age‐related increased iron deposits within regions of the basal ganglia.^40^ We have previously surmised that autonomic dysregulation may influence the inflammatory state in RTT,^64^ and, coupled with the altered subinflammatory profile underlying Rett pathology,^120^ these factors could affect redox‐related physiology in patients. Together with others,^40^ it would therefore be reasonable to presume that the hypoxic conditions caused by the underlying autonomic dysregulation in RTT might cause a redox imbalance that leads to increased iron mineralization. Although autonomic dysregulation and movement disorders are likely to involve different neural pathways, we suggest that buspirone could be one option to consider for the management of severe dystonia in RTT. It would be interesting to see whether reducing breathing dysregulation in RTT using buspirone could indirectly lower redox imbalances and iron accumulation and whether this reduces the clinical severity of dystonia, especially in individuals with truncating mutations who are reported to have a higher frequency of dystonia and rigidity.^22^ Buspirone could also be used alongside free radical scavengers such as vitamin E as there is evidence in animal models of RTT having improved hypoxia tolerance with a vitamin E derivative.^121^

Neuroprotective agents could be other options to consider. Several candidates such as levetiracetam, melatonin, memantine, omega‐3, topiramate, and vitamin E have been examined for their neuroprotective potential.^122^, ^123^ Some of these have been shown to provide neuroprotection in different neurological disorders.^122^ These candidate molecules could delay the progression of movement disorders in rare neurodevelopmental syndromes. Although some of these agents, such as memantine, might offer some promise in RTT,^124^ there is at present no robust empirical evidence to suggest whether these molecules would offer any neuroprotective benefit for patients with RTT. Clinical trials are needed to assess the ability of these molecules to delay motor disease progression in this patient population.

#### Other strategies

Environmental enrichment offers an attractive nonpharmacological strategy to assist in the management of some aspects of movement disorders in RTT. The evidence synthesis showed that even minor improvements in gross and fine motor function would benefit patients. Moreover, environmental stimulation and its positive impact on the individual can also reduce the caregiver burden, as suggested by others.^37^ Environmental and sensory enrichment likely leads to neuroplastic changes caused by upregulation of brain‐derived neurotrophic factor (BDNF) in patients with RTT. This premise is supported by a recent randomized stepped wedge trial in 12 females with RTT who showed improvements in gross motor skills and BDNF levels after 6 months of enriched environmental treatment.^125^ The merits of environmental enrichment should be viewed from the perspective that access to resources among geographical regions will be varied. A Danish study explored the facilitators and obstacles of low‐intensity activities in females with RTT. It highlighted themes that could facilitate health‐promoting interventions in patients with RTT and ways to reduce barriers.^126^ While some evidence suggests that a holistic approach to physical therapy may improve the QoL of individuals with RTT,^127^ the lack of longitudinal follow‐up, methodological weaknesses, and low statistical power^128^, ^129^ necessitates the need to improve the impact of nonmedical intervention among the wider RTT population. Nevertheless, environmental enrichment would be a useful adjunct alongside other interventions for managing the impact of movement abnormalities in patients with RTT.

## Conclusion

Our review emphasizes six key themes. First, it provides a synthesis of clinical features among movement disorders in patients with RTT. Even though some of the clinical features identified are broad and lie across the movement disorder spectrum, under the rubric of clinical care, our information would help clinicians to better understand patterns and trends that emerge. Second, delineating genotype–phenotype relationships can allow for more robust genetic counseling for families and also assist healthcare professionals in monitoring disease progression among different mutations and the potential trajectory of movement disorders. Third, environmental enrichment would be beneficial for improving some aspects of movement disorders. Fourth, the cerebellum and basal ganglia and the dysregulation of the CBGTC loop are likely anatomical targets for movement disorder problems in RTT. Some emerging and adjunct evidence also suggests that increased mineralization of iron within the basal ganglia, particularly within the substantia nigra, putamen, and globus pallidus, correlates with the severity of dystonia in patients with RTT. Fifth, movement disorders can have a variable and sometimes unexpected impact on the QoL of patients with RTT. Finally, the evidence from the systematic review and emerging from the thematic analysis allowed us to describe the developmental trajectory (Table 2) and discuss treatment implications for the management of movement disorders in patients with RTT (Table 3).

Treatment of movement disorders in RTT would require a combined pharmacological and biopsychosocial approach. Recent evidence has demonstrated that presymptomatic training in a mouse model of RTT can help to manage symptoms and delay the onset of the functional impairments.^130^ The translational impact of this work needs to be demonstrated in human studies; however, it strongly suggests that early behavioral training in an individual diagnosed with RTT could delay the onset of motor symptoms. This important finding supports the rationale for routine genetic testing of RTT in newborns.^130^ Building on this work, a proactive approach of environmental enrichment together with pharmacological treatment during the very early stages of the disorder could help in the management of movement disorders, especially in high‐risk individuals where the progression could be quicker. This combined strategy could help patients retain specific aspects of movement and reduce overall disease burden.

## Limitations

While this evidence synthesis is valid, the clinical inferences and their applications to the broader RTT community should be treated with caution because of disparities within the subject area. Different types of movement impairments persist in RTT, and the findings should not be considered universal across the broad spectrum of movement disorders. The severity of illness, co‐occurring comorbidities, and methods used to quantify movement disorders among study groups from different regions limits the generalizability of the findings. The different sample sizes of studies may also influence the findings of genotype–phenotype relationships, especially when examining patient populations with the same mutational profile. Changing definitions^131^ of dystonia/spasticity over time could also be a minor confounder and this could affect diagnostic assessments and the usefulness of existing clinical rating scales. A personalized approach for the treatment and management of movement disorders in RTT is therefore warranted.

A primary search strategy was used that balanced sensitivity and specificity to answer the research questions of the systematic review. We felt that having a broader search term would have resulted in many vague search results and therefore used a more focused primary strategy. Our search strategy, while focused, could have inadvertently omitted some relevant material from the literature. However, by using a secondary search strategy, we were able to extend the reach of our systematic review.

## Disclosure statement

P.S. was a principal investigator (PI) on the Sarizotan (protocol number Sarizotan/001/II/2015; ClinicalTrials.gov identifier: NCT02790034) and GW Pharma (protocol number: GWND18064). P.S. is currently the PI for the Anavex Life Sciences Corp. (protocol number: ANAVEX2‐73‐RS‐002) clinical trial in individuals with RTT. P.S. is the coinventor of the HealthTracker platform, a shareholder in HealthTracker, and the chief executive officer. J.S. has been a trial research methodologist on the Sarizotan Clinical Trial (protocol number Sarizotan/001/II/2015; ClinicalTrials.gov identifier: NCT02790034) and is currently a research manager for the Anavex Life Sciences Corp. clinical trial for individuals with RTT (protocol number: ANAVEX2‐73‐RS‐002). J.S. also advises for Reverse Rett and is on the Reverse Rett Research Review Committee. E.L. and N.N. have no conflicts of interest to declare.

## Author contributions

J.S. conceptualized, drafted, and wrote the systematic review. Both J.S. and E.L. independently searched the databases in a blinded fashion. E.L. independently reviewed the thematic analysis performed by J.S.; and N.N. and P.S. helped provide the clinical translational context of the systematic review. E.L. and P.S. reviewed the intellectual content of the draft and the final versions of the review. N.N. also reviewed the final draft providing specialist expertise on movement disorders. All of the authors approved the final manuscript.

## Availability of data

The data that were used for this systematic review were derived from databases (PubMed, Scopus, Cochrane, PsycINFO, Embase, and Web of Science) that are openly available in the public domain.

## Supporting information


**Supplementary Information S1.** PRISMA flow‐diagram.

**Supplementary Information S2.** Number of records screened using the secondary search strategy. Notes: ^1^There were duplication of records across the databases including articles that were already identified by the primary PRISMA search strategy. ^2^Eighty‐three were trials and one was a Cochrane review.
