# Supplementary figures and images for: Movement disorders in patients with Rett syndrome: A systematic review of evidence and associated clinical considerations
Source: Psychiatry Clin Neurosci. 2021 Oct 21;75(12):369–93. doi: 10.1111/pcn.13299 (PMC9298304; doi:10.1111/pcn.13299)

Supplementary Information 1: PRISMA flow-diagram


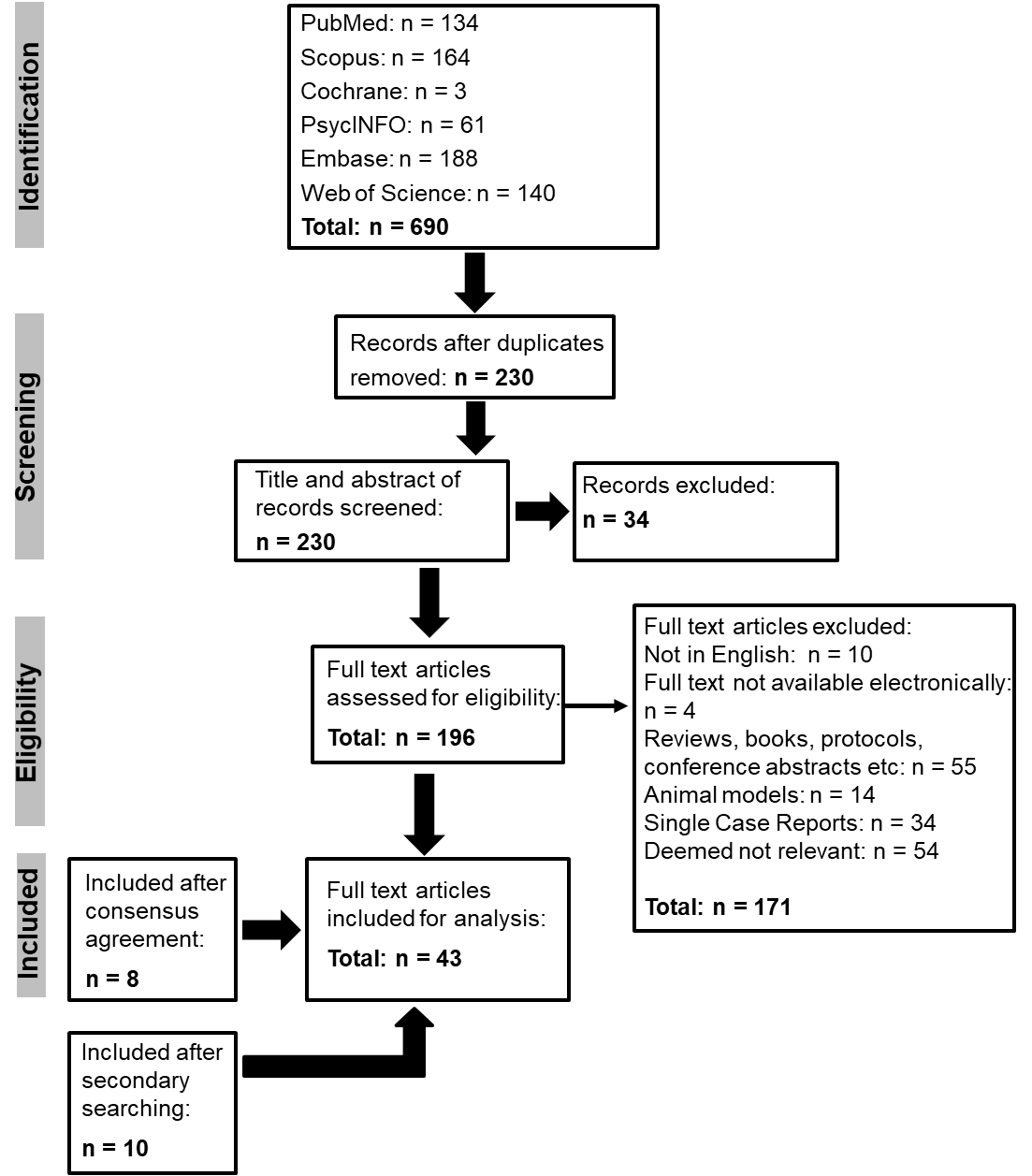

Supplement: Supplementary file 1 — Supplementary Information S1. PRISMA flow‐diagram. [file PCN-75-369-s002.docx]
