# Supplementary material for: Movement disorders in patients with Rett syndrome: A systematic review of evidence and associated clinical considerations
Source: Psychiatry Clin Neurosci. 2021 Oct 21;75(12):369–93. doi: 10.1111/pcn.13299 (PMC9298304; doi:10.1111/pcn.13299)
Supplement: Supplementary file 2 — Supplementary Information S2. Number of records screened using the secondary search strategy. Notes: 1There were duplication of records across the databases including articles that were already identified by the primary PRISMA search strategy. 2Eighty‐three were trials and one was a Cochrane review. [file PCN-75-369-s001.docx]

Supplementary Information 2: Number of records screened using the secondary search strategy

| Search terms | Databases searched | | | | | |
| --- | --- | --- | --- | --- | --- | --- |
|  | PubMed | Cochrane^2^ | Scopus | PsycINFO | Embase | Web of Science |
| (Rett syndrome OR MECP2) AND (dystonia) | 54 | 84 | 53 | 17 | 109 | 76 |
| (Rett syndrome OR MECP2) AND (Parkinson*) | 63 | 84 | 60 | 18 | 257 | 151 |
| (Rett syndrome OR MECP2) AND (bruxism) | 38 | 84 | 60 | 12 | 97 | 42 |
| (Rett syndrome OR MECP2) AND (spasticity) | 85 | 84 | 60 | 5 | 139 | 81 |
| (Rett syndrome OR MECP2) AND (tremor*) | 29 | 84 | 42 | 12 | 81 | 49 |
| (Rett syndrome OR MECP2) AND (ataxia*) | 149 | 84 | 145 | 24 | 263 | 216 |
| Number of additional articles identified^1^ | 6 | 0 | 1 | 2 | 1 | 0 |

Notes:

^1^There were duplication of records across the databases including articles that were already identified by the primary PRISMA search strategy.

^2^Eighty-three were trials and one was a Cochrane review.
